# Laparoscopic bridging vs. anatomic open reconstruction for midline abdominal hernia mesh repair [LABOR]: single-blinded, multicenter, randomized, controlled trial on long-term functional results

**DOI:** 10.1186/1745-6215-14-357

**Published:** 2013-10-28

**Authors:** Cesare Stabilini, Umberto Bracale, Giusto Pignata, Marco Frascio, Marco Casaccia, Paolo Pelosi, Alessio Signori, Tommaso Testa, Gian Marco Rosa, Nicola Morelli, Rosario Fornaro, Denise Palombo, Serena Perotti, Maria Santina Bruno, Mikaela Imperatore, Carolina Righetti, Stefano Pezzato, Fabrizio Lazzara, Ezio Gianetta

**Affiliations:** 1 Department of Surgical Sciences (DISC), University of Genoa, Genoa, Italy; 2 Department of General Vascular and Thoracic Surgery, University of Naples “Federico II”, Naples, Italy; 3 General and Minimally-Invasive Surgical Unit, San Camillo Hospital, Trento, Italy; 4 Department of Health Sciences (DISSAL), University of Genoa, Genoa, Italy; 5 Department of Internal Medicine (DiMI), University of Genoa, Genoa, Italy; 6 Casa du Cura Villa Igea, Acqui Terme, Alessandria, Italy

**Keywords:** Incisional, Midline, Hernia, Laparoscopy, Mesh, Functional results, Rives, Randomized

## Abstract

**Background:**

Re-approximation of the rectal muscles along the midline is recommended by some groups as a rule for incisional and ventral hernia repairs. The introduction of laparoscopic repair has generated a debate because it is not aimed at restoring abdominal wall integrity but instead aims just to bridge the defect. Whether restoration of the abdominal integrity has a real impact on patient mobility is questionable, and the available literature provides no definitive answer. The present study aims to compare the functional results of laparoscopic bridging with those of re-approximation of the rectal muscle in the midline as a mesh repair for ventral and incisional abdominal defect through an “open” access. We hypothesized that, for the type of defect suitable for a laparoscopic bridging, the effect of an anatomical reconstruction is near negligible, thus not a fixed rule.

**Methods and design:**

The LABOR trial is a multicenter, prospective, two-arm, single-blinded, randomized trial. Patients of more than 60 years of age with a defect of less than 10 cm at its greatest diameter will be randomly submitted to open Rives or laparoscopic defect repair. All the participating patients will have a preoperative evaluation of their abdominal wall strength and mobility along with volumetry, respiratory function test, intraabdominal pressure and quality of life assessment.

The primary outcome will be the difference in abdominal wall strength as measured by a double leg-lowering test performed at 12 months postoperatively. The secondary outcomes will be the rate of recurrence and changes in baseline abdominal mobility, respiratory function tests, intraabdominal pressure, CT volumetry and quality of life at 6 and 12 months postoperatively.

**Discussion:**

The study will help to define the most suitable treatment for small-medium incisional and primary hernias in patients older than 60 years. Given a similar mid-term recurrence rate in both groups, if the trial shows no differences among treatments (acceptance of the null-hypothesis), then the choice of whether to submit a patient to one intervention will be made on the basis of cost and the surgeon’s experience.

**Trial registration:**

Current Controlled Trials ISRCTN93729016

## Background

Incisional hernia is one of the most common complications following abdominal surgery, with a reported incidence of 3-13% [1,2]. It is evident from recent trials that mesh adoption for the repair of these abdominal wall defects has dramatically reduced the recurrence rate after surgery in comparison with traditional simple suture repair [3,4]. Nevertheless, the best technique for the repair of an incisional hernia to date has yet to be defined. In fact, a recent meta-analysis on the open repair of incisional hernias [5] was not able to demonstrate the superiority of any of the available repairs independently from the mesh placement in the abdominal wall (onlay, sublay and inlay), the type of mesh used (lightweight, heavyweight) or the addition of anatomical dissection aimed at increasing abdominal compliance (i.e., component separation).

These findings could support the following hypotheses:

•The good results, in terms of recurrence, obtained with each technique could have a key point in the adoption of a mesh.

•The superiority of one technique over another should be based on outcomes other than hernia recurrence.

Very little evidence [6,7] and recently a large prospective trial [8] comparing laparoscopic and open techniques in terms of a quality of life (QOL) measurement tool specific for hernia outcome study (the Carolina Comfort Scale) have indicated a possible benefit in the short term granted by the open fascial closure of the abdominal defect. Basing their assumption on these observations and on the equivocal results on QOL from trials comparing laparoscopic and open ventral hernia repair [9,10], experts in the field [11,12] have hypothesized that medialization of the rectus abdominis muscles improves abdominal wall function. This speculation has not been objectively proven because measures of the recurrence rate, quality-of-life scores and patient questionnaire responses remain the only established criteria for assessing abdominal wall function after hernia repair. Nevertheless, expert groups recommend this approach for ventral hernia repair as a rule to centralize and re-approximate the rectus muscles along the midline to the extent possible. This step attempts to restore the functional, innervated abdominal wall and perform dynamic reparation without undue tension. Commonly used techniques with the aforementioned characteristics that have been used for the repair of abdominal hernias include retrorectus (i.e., the Rives-Stoppa procedure) and component separation (the Ramirez technique). Thus, several surgeons consider these the current “gold standard” treatments.

Based on this assumption, even if laparoscopic surgery has gained an adequate scientific consensus for the surgical treatment of different diseases [13,14], certain criticisms have been raised over its introduction in the routine practice of abdominal hernia repair. These techniques, which avoid the restoration of anatomical integrity for a simple bridging of the defect, appear “less effective.”

Nevertheless, a recent systematic review of studies comparing laparoscopic and open techniques [15] was not able to confirm this assumption. On the contrary, the review indicated that laparoscopic incisional hernia repair is generally safe, with a lower risk of wound infection and shorter hospital stays relative to open surgery.

Surprisingly and in opposition to previous studies included in the Cochrane review, a recently published randomized trial [16] indicated that postoperative pain and recovery at 3 weeks is not affected by the type of adopted hernia repair, but the laparoscopic approach results in better physical function than the open repair based on SF36 subscale values. This observation is only partly the consequence of the reduced number of surgical site infections following laparoscopy. Moreover, these findings could support the hypothesis that procedures aimed at restoring the anatomy that involve extensive and potentially damaging dissection in an already scarred myoaponeurotic region could be less effective for the patient. In other words, in certain circumstances, anatomic reconstruction could be an excessive burden for an already compromised abdominal wall. Nevertheless, a self-reported questionnaire cannot be accepted as the sole explanation of this phenomenon, which should be objectively investigated and measured.

To obtain an answer to the debate, an emerging type of analysis is being more frequently used: the functional evaluation of the motility and muscular strength of the abdominal wall. The modalities to evaluate the trunk flexor muscles and the strength of the abdominal wall are isokinetic dynamometer measurement and physical testing.

The isokinetic dynamometer (Biodex Model 2000, Multijoint System 3, Biodex Corp., Shirley, NY, USA) is a safe way to load a dynamically contracting muscle to maximum capability throughout its entire range in a continuous motion and to evaluate the trunk flexor strength during isokinetic movement at a constant angular velocity. An inexpensive alternative to using a machine for evaluation is the use of physical tests. The most commonly used tests are the double leg-lowering test (DLL) and the trunk-raising test (TR). Both of them explore the strength of the rectal muscle, asking the patient to perform some curl-type exercises. After categorization of DLL and TR, it is possible to create an abdominal wall strength score (AWS) with a minimum score of 0 and a maximum of 10 points.

Thus far, the aforementioned instruments have been used in two published studies without resolving the debate over the best intervention between bridging and reconstruction of the abdominal wall. The dynamometer was used in a study by den Hartog and coworkers [17] in which trunk flexor strength was compared for a group of 30 patients submitted to laparoscopic or open hernia repair with rectal approximation and a group of 12 healthy subjects. The mean torque/weight (N m/kg) for trunk flexion was significantly higher in the control group compared with that of the incisional hernia repair patients. Moreover, among the patients who underwent surgery, a significantly higher peak torque/weight was observed in the open technique group compared with the laparoscopic technique group. Based on these measures, the authors considered the two-layered technique more effective in repairing the abdominal wall of patients with incisional hernias and even more protective against some types of complication (back pain) in comparison with laparoscopic bridging. The multiple limitations represented by the fact that the study was not prospective and largely underpowered and that the patients in the open surgical group were submitted to a repair without mesh make the results not completely reliable.

The data for the physical testing method appear to be more interesting. The data available for the DLL and TR tests and combined AWS score are currently validated in clinical practice [18], but results for incisional hernia are still not available. The adoption of this type of test to evaluate the results of incisional and ventral hernia repair could be interesting because such results would aid in discerning whether, hypothetically, the advantage granted by anatomical reconstruction over laparoscopic bridging is real and the data provided by the dynamometer can be translated into clinical practice. The hypothesis of the study is that, for abdominal ventral defects suitable for a laparoscopic repair (10 cm at major dimension), the bridging technique is similar to the open reconstruction in terms of restoration of abdominal wall function. We believe that the best way to demonstrate this hypothesis is by measuring the differences among these groups in a clinical variable with a real impact for the patients (physical testing, i.e., DLL, TR, AWS score). The results of the physical testing will be correlated with clinical parameters (respiratory function tests, intraabdominal pressure, CT volumetry and quality of life) in order to better address the physiological implications of each of the repair techniques.

### Aim of the study

The aim of this study is to define the role of anatomical reconstruction and simple defect bridging in the mesh repair of incisional and primary ventral hernias. We wanted to analyze in depth whether defect bridging is comparable to anatomical reconstruction with mesh in terms of the strength of the abdominal wall as expressed by the differences among groups 1 year postoperatively in the grades of leg extension over the trunk during a DLL test.

## Design

LABOR is a randomized, controlled, non-inferiority trial. Patients will be randomly allocated to receive a mesh repair of their incisional hernia with either laparoscopic bridging or open anatomical reconstruction. The trial has been registered on Current Controlled Trials as ISRCTN93729016.

### Sample size calculation, randomization and statistical analysis

On the basis of a previous published work, the minimum DLL to define the non-inferiority of laparoscopic bridging vs. anatomic open reconstruction will be set to 5 degrees with a standard deviation of 8.5 degrees. With this hypothesis and for a power of 90% and an alpha of 5%, we will need 50 patients in each arm (total 100). If we consider a possible dropout rate of 10%, a total of 110 patients would be sufficient. As a consequence, approximately 20 patients for each center will be required.

A centralized block randomization stratified for center will be performed by a statistician not involved in patient management. Blocks will have a variable dimension (random) between 4 and 12 units. A total of five blocks will be obtained for each center in the event that the investigator continues enrollment beyond the initially planned sample size. The local investigators, after obtaining written informed consent of patients, will receive the randomization arm from the statistical center for each patient, then the patient will be allocated to the treatment group as indicated.

Data analysis will be performed by a statistician not involved in data collection at the main investigation center (University of Genoa). The means and standard deviations or medians with ranges, if asymmetry of data is detected, will be reported for all continuous demographic and clinical characteristics recorded, whereas counts and percentages will be recorded for categorical characteristics (e.g., gender, functional status and smoking habits).

To compare the DLL test results during follow-up between the two groups of treatment, ANOVA for repeated measures will be adopted using DLL as the dependent variable. Because the DLL test cannot have a normal distribution, a ranking transformation will eventually be used. The same approach will be used for the other ordinal characteristics used as secondary outcomes (VAS as scoring for pain and AWS score) and, in general, for all other outcomes considered. If a consistent number of missing values is detected during the follow-up, ANOVA for repeated measures will be replaced by linear mixed or generalized estimating equation (GEE) models, which permit the exclusion of the data only from a single missing observation and not from the patient on overall as with ANOVA. Single comparisons between baseline values and each time during follow-up will be assessed using Bonferroni correction for multiple comparisons. All analyses will be performed on an intention-to-treat basis. A *p*-value lower than 0.05 will be considered statistically significant. SPSS (v.20, IBM Corp., Armonk, NY, USA) will be used for the statistical analyses.

### Participants

The researchers will recruit consecutive patients who are affected by an incisional hernia scheduled for surgical repair and meet the criteria for enrollment over a period of approximately 1 year. The patients in the participating units will be screened daily. Demographic data on screened patients, regardless of meeting enrollment criteria, will be recorded (registry: age, gender and type of surgery). We will randomize 110 patients admitted into the participating centers. It is expected that each participating center will randomize at least 20 patients who will meet all of the inclusion criteria.

Based on the current literature [19], it was decided to enroll patients with a defect suitable for both laparoscopic and open repair. For this reason, we have set the upper limit of the defect size at a width of 10 cm. We decided to set 60 years old as the cutoff age because that is the mean age in recent high-quality, randomized studies [4], and this cutoff value will minimize the difference in trunk flexor strength observed in previously cited papers and generated by differences in age [17].

### Inclusion criteria

Patients will be eligible for study enrollment if they meet the following criteria:

(1) Presence of a midline incisional or a ventral primitive hernia [15]

(2) Dimension of the defect measured on preoperative CT scan is as follows:

(a) Primary ventral hernia ≥4 to ≤10 cm at its greatest diameter according to a preoperative CT scan (“large” according to EHS classification for primary ventral hernia) [20]

(a) Incisional hernia ≥4 to ≤10 cm at its greatest diameter according to a preoperative CT scan (W2 according to EHS classification for incisional hernias [20])

3 Both genders

4 ≥60 years of age

5 Provide informed consent for randomization

### Exclusion criteria

1. Patients with non-midline defects or diastasis recti without herniation

2. BMI ≥ 35 kg/m^2^

3. Hernia with a previous mesh repair attempt

4. Patients classified as American Society of Anesthesiologists class 4 or 5

5. Patients with a severe comorbid condition likely to limit survival to 2 years

6. Patients with cirrhosis with or without ascites

7. Patients under immunosuppressive treatment

8. Patients with acute bowel obstruction, strangulation, peritonitis or perforation

9. Presence of local or systemic infection

10. Patients with neuromuscular disease likely to impair motility (e.g., previous ictus with reliquate)

11. Patients refusing to participate in the study

### Setting

Patients will be enrolled at six different surgical units across Italy, tertiary referral centers for both laparoscopy and abdominal wall surgery (three university teaching hospitals). The involved surgeons are experienced in the field of abdominal wall reconstruction and advanced laparoscopic techniques and have treated at least 20 cases for each trial procedure [21].

At least two investigators from each participating center will be involved in the study. One researcher will receive the allocation treatment for the patient after calling the statistician of the main investigation center (University of Genoa). The other investigator, blinded to the randomization arm, will score the primary and secondary postoperative endpoints.

### Screening and recruitment

Patients in the participating units will be screened daily. Demographic data on screened patients, regardless of meeting enrollment criteria, will be recorded (registry: age, gender and type of surgery). If the patient is determined to be eligible according to the study criteria, he/she will be submitted to a CT scan of the abdomen without contrast media. If the parameters of the defect are suitable, then the patient will be enrolled.

### Patient consent

According to local regulations, all patients or legal representatives have to provide written informed consent before inclusion in the study. Patients will be asked to sign the consent form specific to the type of surgery performed as well as the form for randomization. Every patient will receive a letter to inform his/her general practitioner of enrollment in the study.

### Blinding

Every patient will be informed that he/she will undergo a mesh repair of the abdominal wall defect and will be informed of the type of approach used (laparoscopic or open), but he/she will not be informed of the type of repair adopted (reconstruction or bridging) nor of the type of hypothetical advantages of the technique. The patient will be informed of the complications, recurrence rate and sequelae of a typical incisional hernia repair with mesh.

Postoperatively, the examiner executing the physical and QOL-related tests will be blinded to the type of surgery received by the subjects. He/she will be a resident or physician who was not involved in the operation and will examine the patient with dressings on. During the hospital stay, the patient will have a wide dressing on the surgical wound.

### Interventions: surgery

The patient will be divided into two groups according to the randomized procedure:

Group R (reconstruction)

The planned number of patients will be submitted to a Rives-Stoppa procedure for the repair of the defect [22].

Group B (bridging)

In this group, after randomization patients will be operated on using a standard laparoscopic technique [23].

### Mesh

To minimize the effect due to different materials and density, every center will be encouraged to use the same type of mesh. A composite, lightweight, polypropylene mesh suitable for both retromuscular and intraperitoneal repair should be adopted.

### Fixation methods

The mesh will be sutured in Group R with standard, non-resorbable passing sutures. In Group B, absorbable tacks placed with the double crown technique and reinforced with fascial sutures will be used.

### Perioperative management

#### Before surgery

Patients will be admitted the day before or on the day of surgery. Patients under antiplatelet and warfarin therapy will be instructed to stop therapy 7 days before surgery. Low-molecular-weight heparin will be administered according to thrombosis risk. No bowel preparation will be required. According to national guidelines, the patients will be submitted to a single dose of a first- or second-generation cephalosporin (e.g., cefazolin 2 g) as antibiotic prophylaxis at the moment of induction of anesthesia. This dose will be repeated according to the duration of surgery [24]. In cases of different bacterial or high-risk settings, the final decision on antibiotics will be left to each individual surgeon.

### Surgical period

Group B will not use any surgical drain (laparoscopic group). Group R will be allowed to use drains according to the surgeon’s discretion [25]. Patients in group B will receive a local anesthetic for each trocar insertion site (ropivacaine HCl, 0.75%, 5 ml each). In group R, the patients will receive anesthetic in the whole wound (ropivacaine HCl, 0.75%, 15 ml). A bladder catheter will be inserted in the ward to assess intraabdominal pressure with a transducer. The catheter will be removed on the first postoperative day after the final measurement of intraabdominal pressure.

### Postoperative

Postoperative pain control is planned with the elastomeric pump infusion of painkillers. Parenteral opioids will be used for rescue analgesia. Patients will be allowed liquids on the night of surgery, a semisolid diet on the first postoperative day and a light meal on the second. In case of unplanned resection, the decision will be left to operating surgeon. Patients will be discharged when they are conscious and orientated, able to tolerate a solid diet, have regular bowel functions and are able to take care of themselves.

Postoperative medications:

Low-molecular-weight heparin will be prescribed for deep venous thrombosis and pulmonary embolism prophylaxis. Painkillers and antibiotics will be prescribed based on the patient’s needs. Patients will also be prescribed a post-surgical belt for 30 days to minimize seroma formation.

### Follow-up

The patient will be submitted to a 1-year postoperative follow-up. Planned follow-up visits will be on the following days:

•10 ± 3 days (wound evaluation and clinical evaluation)

•30 ± 3 days (clinical evaluation and study test performance)

•180 ± 3 days (clinical evaluation and study test performance)

•360 ± 3 days (clinical evaluation and study test performance)

### Outcomes

#### Primary outcome

Changes in baseline DLL measures at 12 months postoperatively in both groups.

#### Secondary outcome

Among the subjects in each group of treatment, the secondary outcomes are as follows:

•differences in DLL (preoperation and at 1 and 6 months postoperation)

•differences in TR test (preoperation and at 1, 6 and 12 months postoperation)

•AWS score (preoperation and at 1, 6 and 12 months postoperation)

•respiratory function tests (preoperation and at 6 and 12 months postoperation)

•intraabdominal pressure (preoperation and postoperation)

•pain

•quality of life (SF36, preoperation, postoperation, 1 month postoperation, 6 months postoperation and 12 months postoperation)

•recurrence rate in each group

### Performance and measures: double leg-lowering test (DLL: how and when)

A. In a supine position, the patient raises both legs; the movable arm of a goniometer is held tightly against the longitudinal axis of the patient’s lateral thigh by the examiner’s hand. As the patient lowers the legs, the examiner notes the angle between the extended legs and the table at the moment the pelvis tilts anteriorly and the lower back arches off the table. This angle is subtracted from 90° to determine the angle of interest.

**Figure 1:**
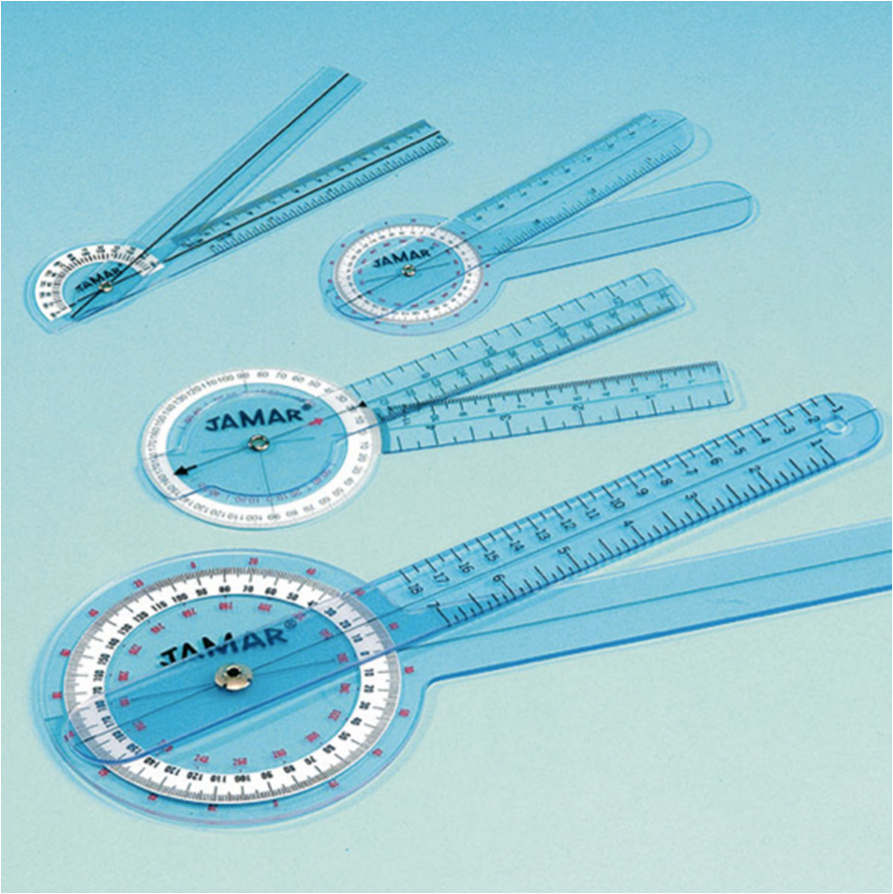
Jamar E-Z Read Goniometer 12/32 cm.

The instrument used for the DLL test is the Jamar E-Z Read Goniometer 12/32 cm (Figure 1), which has a scale from 0° to 180° and from 0° to 360° in 1° increments (inch and centimeter linear measurements). The instrument is made with transparent plastic, making it easy to observe the joint as it moves through the range of motion.

To obtain a more precise and less variable measurement, before performing the test, three colored dots will be applied to the hip and ankle of the patients and on the underlying surface. During the test, a picture of the patient will be shot at maximum leg rise. Using the dots as reference points, the angle will be measured with computer software directly on the picture.

After measuring the angle, a score will be assigned for the calculation of AWS (5 points).

B. The test will be performed preoperatively and at 1 month, 6 months and 12 months postoperatively.

•41° or more (normal) 5 points

•31°-40° (good) 4 points

•21°- 30° (fair) 3 points

•11°-20° (poor) 2 points

•0°-10° (trace) 1 point

### Performance and measures: trunk-raising test (TR: how and when)

A. The TR test does not require any instruments. The examiner evaluates whether the supine patient (hips flexed 45° and knees at 90°) is able to perform some simple exercises that are scored for points (maximum 5 points). The score for this test is determined by the arm position the patient uses to elevate the scapulae off the table and how long the patient is able to hold this position.

••Hands behind neck, scapulae clearing the table, 20” hold (normal), 5 points.

••Arms crossed over the chest, scapulae clearing the table, 20” (good), 4 points

••Arms straight, scapulae clearing the table, 10” hold (fair), 3 points

••Arm extended toward the knees, top of scapulae lifting from the table (poor), 2 points

••Inability to raise more than the head off the table (trace), 1 point

B. The test will be performed preoperatively and at 1 month, 6 months and 12 months postoperatively.

### Abdominal wall strength (AWS) score

A. We will obtain the AWS score by summing the results of the DLL and TR tests. The AWS score ranges from 0 to 10 points.

B. The test will be performed preoperatively and at 1 month, 6 months and 12 months postoperatively.

### Quality of life

A. It appears appropriate for the purpose of the study to use the Medical Outcome Study Short Form-36 (SF-36) Health Survey. This is a comprehensive assessment of overall health status encompassing physical, emotional and mental life aspects over a predefined period of time [26,27]. It is a multidisciplinary questionnaire composed of 36 items that determine the health-related quality of life [28]. Before surgery, the generic quality of life will be prospectively measured using the Medical Outcome Study SF-36 Health Survey, which is composed of eight different health-quality domains: physical and social functioning (PF and SF, respectively), body pain (BP), general health perception (GH), physical and emotional role limitations (RP and RE, respectively), vitality (VT) and mental health (MH). The scores for each domain range from 0 to 100, with higher scores indicating better quality of life.

The score has been already extensively validated and adopted in clinical practice and in the field of abdominal wall hernia surgery [16,29,30].

B. The SF36 will be administered preoperatively and at 1 month, 6 months and 12 months postoperatively.

### Scoring for pain

A. A visual analog scale (VAS) will be used for patient pain evaluation. As the name implies, VAS uses an analog format, meaning that it represents a continuous range of values [31,32]. The most common style used in pain measurement uses a horizontal line measuring exactly 10 cm (100 mm). The patient is asked to make a mark on this line, and then the line is measured and recorded in millimeters or centimeters (e.g., 37 mm or 3.7 cm). The length of the line is important for this outcome measure because this tool has been evaluated in this format, and the measurement relies on the line being exactly 10 cm long. The scale is formatted without numbers.

B. Measures of VAS will be taken preoperatively, postoperatively on day 1, the day of discharge and 10 days, 6 and 12 months postoperatively.

### Performance and measures: intrabdominal pressure (IAP: how and when)

A. Patients will be submitted to evaluation of intraabdominal pressure (IAP) according to current guidelines [33]. IAP is the pressure concealed within the abdominal cavity and should be expressed in mmHg and measured at end-expiration in the complete supine position after ensuring that abdominal muscle contractions are absent and that the transducer is zeroed at the level of the mid-axillary line. The reference standard for intermittent IAP measurement is via the bladder with a maximal instillation volume of 25 ml of sterile isotonic saline (normal IAP is approximately 5–7 mmHg in critically ill adults).

B. Patient IAP will be assessed preoperatively, at the end of the surgical procedure (curarized) and on the first postoperative day, before catheter removal.

### CT scan

A. Patients will be submitted to a CT scan to evaluate the size of the defect and decide the final enrollment into the study in the preoperative phase. The CT requirement is a scan able to produce images with 1.2 isometric voxels.

B. CT scans will be performed preoperatively and 12 months postoperatively to rule out recurrences, evaluate interrectal distances and evaluate the volumetry of the abdominal cavity with correlation to preoperative values.

### Respiratory function evaluation

A. Pulmonary function tests will be interpreted by a pulmonologist who is blind to the study and its participants. Forced vital capacity (FCV) and forced expiratory volume (FEV1) will be measured. Intraoperative peak airway pressures, measured at the end of the inspiratory phase of each breath, will be automatically calculated by the mechanical ventilator and recorded by the anesthesiologist prior to opening the abdomen, while the abdomen is open, after rectus muscle plication and after skin closure.

B. Spirometry will be performed on all patients during the preoperative visit and then 6 or 12 months postoperatively.

### Recurrence

A. Recurrence is defined as any postoperative bulging in the area of the edge of the mesh.

B. At each postoperative visit, each patient will be examined by an independent surgeon to determine the presence or absence of recurrence. Recurrences detected clinically will be confirmed by computed tomography of the abdomen or during a second operation.

### Postoperative complications definition and grading

To evaluate postoperative complications and standardize their reporting, we decided to use the classification introduced by Dindo and coworkers in 2004 [34].

### Data collection

Data will be gathered at each single unit for each single subject by filling out the provided Case Report Form (CRF). The original CRFs of all participants will be kept in the unit in which the patients undergo the surgical procedure. A copy of the CRF will be transmitted (either scanned and e-mailed or copied and mailed by traditional mail) to the University of Genoa Department of Surgery and Integrated Diagnostics (DiSC). After data collection, all CRFs will be sent for data extraction and interpretation to the Department of Biostatistics, University of Genoa.

### Ethical aspects

The study was approved by the local Institutional Review Board (Comitato Etico, IRCCS San Martino IST, protocol no. 2/2013). The study is performed in agreement with the principles of the Declaration of Helsinki. All patients are informed about the purpose and the risks of the study and about their right to withdraw their consent at any time. Patients are only included in the study if written informed consent has been given.

## Discussion

The question of which technique should be considered the gold standard for the repair of incisional hernia remains unanswered. Currently, the topic of hernia recurrence has declined in importance because of the adoption of mesh repair, but the question of which procedure best suits the needs of the patients undergoing surgery remains open. Before a definitive conclusion can be drawn, it is imperative to correctly investigate the physiological effects of the type of repair adopted. Some premises must be considered when facing this issue. First, the patient must be considered to be a typical case with an incisional hernia and must be in their 6th or 7th decade of life. Patients at this age currently have an increased life expectancy and experience a more active lifestyle. Thus, a procedure aimed at restoring abdominal wall integrity and function may be better able to fulfill the needs of this group.

In addition, the physiological changes associated with aging have an important effect on abdominal muscles. In this period, even if not maximal, a process of lean body mass reduction starts with the impairments of mobility and total muscular strength [35]. This muscular hyposthenia could also be hampered by muscle fibrosis and atrophy occurring after incisional hernia development. This process has been recently outlined in animal models, and it is similar to what is observed in mechanically unloaded muscles [36].

The possibility of muscular structure reversal after these myopathic changes have occurred is interesting [37]. Moreover, Culbertson et al. demonstrated that a tension-free repair with mesh (bridging technique) in which the injured muscle is partially reloaded induces a better recovery of muscular structure in comparison with that of a simple repair with tension. The group postulated that “excessive tension may delay muscle recovery so there may be an optimal range of muscle length and tension during repair that results in sufficient reloading forces, while avoiding excessive tension that may delay recovery.” This interesting observation could lead to the adoption of simple bridging techniques or a hybrid Rives-Ramirez procedure in which the stability of the repair is provided by the mesh and the tension is reduced by myofascial releasing incisions.

Moreover, with the rapidly increasing use of laparoscopy, the numbers and size of incisional hernias have been reduced. Consequently, it can be postulated that two types of abdominal wall defects are currently more frequently encountered: small (<10 cm in their major diameter) primitive and incisional hernias and giant abdominal hernias. Given the aforementioned hypotheses, a patient with a small defect would receive an advantage from a simple bridging of the defect with mesh. This effect would be better indicated in comparison with an anatomical reconstruction for the inferior burden in terms of dissection, the reduced operative time and the possibility of using a less invasive approach. In contrast, a giant abdominal defect has a larger impact on the anatomy, abdominal wall compliance and the personal image of the patient. Moreover, the predicted muscular damage to the abdominal wall could be relevant. Future research needs to be conducted to determine whether, in these cases, a simple bridging would be equivalent or superior to an anatomical reconstruction and to evaluate the effects on abdominal wall function recovery.

We decided to adopt the outcome of DLL because it represents a more straightforward method to define the ability of a procedure to restore abdominal wall function. In fact, we have considered that a dynamometer is the ideal way to measure a difference because it offers a quantitative calculation of a variable that can be compared among different groups. However, as occurs for other medical variables, a significant difference could be clinically irrelevant.

### Clinical implications

Given the constant and equal number of recurrences in both groups, if patients submitted to laparoscopic repair have the same results in physical testing in comparison with open repair, then the trial would indicate (*acceptance of the null hypothesis*) that an anatomic repair of incisional hernia through rectal muscles medialization does not offer any advantage to the patient. The reasons for this deduction could be twofold: first, the anatomic repair is not effective on previously scarred tissue, and, second, the dimensions of the defect included in this trial are too small to determine a perturbation of the abdominal motility. The clinical implications for these results would be that the laparoscopic bridging hernia repair in patients over 60 years of age should be considered a valid alternative to open repair, for both its efficiency and functional, or even superior, results given the advantages already demonstrated in previous trials (fewer wound complications, shorter postoperative hospital stay). It would remain an open question whether the restoration of the anatomic abdominal structure would be useful in younger patients. If the results reveal a better test performance in the anatomic reconstruction group (*rejection of a null hypothesis*), the choice of this type of intervention as the gold standard technique would be supported by clear evidence of an advantage offered in terms of the functional restoration of the abdominal wall.

## Trial status

Patient enrollment started in June 2013.

## Abbreviations

DLL: Double leg lowering test; TR: Trunk raising test; AWS: Abdominal wall strength score; IAP: Intraabdominal pressure; LMWH: Low molecular weight heparin; DVT: Deep venous thrombosis” respectively; QOL: Quality of life.

## Competing interests

The LABOR study will be completely funded by university and hospital resources. The authors declare no financial competing interests. There are no non-financial competing interests.

## Authors’ contributions

CS conceived the study, is responsible for bibliographic searching and screening, revised the case report form model and chose the test types, is responsible for manuscript drafting and participated in the critical revision of the manuscript. UB conceived the study, participated in bibliographic screening, provided surgical and laparoscopic supervision, and participated in the drafting and critical revision of the manuscript. GP conceived the study, provided surgical and laparoscopic supervision, and participated in the drafting and critical revision of the manuscript. FM participated in the drafting and critical revision of the manuscript. MC supervised ethical issues and participated in the critical revision and drafting of the manuscript. PP conceived the study, designed the study, provided anesthesiologic and respiratory counseling, and participated in the critical revision of the manuscript. AS conceived the study, designed the statistical analyses, and will be responsible for patient randomization and data analyses. TT provided surgical technical supervision and participated in the critical revision of the manuscript. GMR provided medical counseling, chose test types, and participated in the drafting and revising of the manuscript. NM provided surgical technical supervision and participated in the critical revision of the manuscript. RF participated in the bibliographic screening and in the critical revision of the manuscript. DP conceived the study, conceived and prepared the case report form model, chose test types, and participated in the drafting and critical revision of the manuscript. SP conceived the study, conceived and prepared case report form model, chose test types, and participated in the drafting and critical revision of the manuscript. MSB is responsible for bibliographic searching and screening, and participated in the drafting and critical revision of the manuscript. MI is responsible for bibliographic searching and screening, and participated in the drafting and critical revision of the manuscript. CR is responsible for bibliographic searching and screening, and participated in the drafting and critical revision of the manuscript. SP is responsible for bibliographic searching and screening, and participated in the drafting and critical revision of the manuscript. FL is responsible for bibliographic searching and screening, and participated in the drafting and critical revision of the manuscript. EG conceived the study, is responsible for bibliographic screening, revised the case report form model and chose test types, is responsible for manuscript drafting and participated in the critical revision of the manuscript. All authors read and approved the final manuscript.
